# CoCrFeNi High-Entropy Alloy as an Enhanced Hydrogen Evolution Catalyst in an Acidic Solution

**DOI:** 10.1021/acs.jpcc.1c03646

**Published:** 2021-08-03

**Authors:** Frank McKay, Yuxin Fang, Orhan Kizilkaya, Prashant Singh, Duane D. Johnson, Amitava Roy, David P. Young, Phillip T. Sprunger, John C. Flake, William A. Shelton, Ye Xu

**Affiliations:** † Department of Physics and Astronomy, Louisiana State University, Baton Rouge, Louisiana 70803, United States; ‡ Cain Department of Chemical Engineering, Louisiana State University, Baton Rouge, Louisiana 70803, United States; § Center for Advanced Microstructures and Devices, Louisiana State University, Baton Rouge, Louisiana 70803, United States; ∥ United States Department of Energy, Ames Laboratory, Ames, Iowa 50011, United States; ⊥ Department of Materials Science and Engineering, Iowa State University, Ames, Iowa 50011, United States

## Abstract

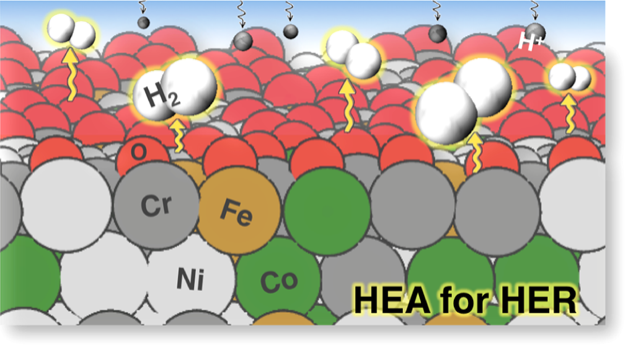

High-entropy alloys (HEAs) have intriguing material properties, but their potential as catalysts has not been widely explored. Based on a concise theoretical model, we predict that the surface of a quaternary HEA of base metals, CoCrFeNi, should go from being nearly fully oxidized except for pure Ni sites when exposed to O_2_ to being partially oxidized in an acidic solution under cathodic bias, and that such a partially oxidized surface should be more active for the electrochemical hydrogen evolution reaction (HER) in acidic solutions than all the component metals. These predictions are confirmed by electrochemical and surface science experiments: the Ni in the HEA is found to be most resistant to oxidation, and when deployed in 0.5 M H_2_SO_4_, the HEA exhibits an overpotential of only 60 mV relative to Pt for the HER at a current density of 1 mA/cm^2^.

## Introduction

Platinum-group metals are used in a variety of critical catalytic processes in petrochemical, automotive, pharmaceutical, and other industries. Due to high costs and limitations on supplies, both the United States and the EU have placed Pt-group metals on critical materials lists,^1,2^ seeking their replacement by more earth-abundant materials in the near future.^3^ High-entropy alloys (HEAs) are known to exhibit a range of interesting material properties including high yield strengths, fracture resistance, and superconductivity.^4−7^ Recently, moreover, HEAs have captured the attention of catalysis researchers.^8−12^

Alloying has long been practiced to modify the catalytic performance.^13^ Yet, the field of catalysis has largely dwelled on binary alloys or well-defined structures (e.g., intermetallics and core–shell particles).^14−17^ It has not ventured far with multimetallic alloys (MMAs), which offer a large, mostly unexplored design space beyond bimetallic systems where compositions may exist that mimic precious metal catalysts. However, the complexity of MMAs has hindered the understanding of their surface reactivity at the molecular level and the scientific progress in MMA catalysis.

Alloying 3d base metals, including Co, Cr, Fe, and Ni, with Pt has been studied as a way to reduce the use of Pt and enhance its activity in low-temperature fuel cells, but the presence of Pt remains indispensable to desired functionality.^18−20^ Related alloys (e.g., Ni–Co,^21^ Ni–Fe–Mo–Co–Cr,^11^ and Co–Cr–Fe–Ni–Al^22^) have been tested for hydrogen evolution reaction (HER) without fundamental insights into the chemical origin of their activity. Here, we predict, through a theoretical analysis of the surface reactivity of this HEA using an approximate surface model^23,24^ instead of a parameterization approach,^9,12,25,26^ that one particular HEA, CoCrFeNi, shows activity for the electrochemical HER that is closer to that of Pt than all of the individual component metals. This is then confirmed through electrochemical testing of the HEA in a 0.5 M aqueous solution of H_2_SO_4_. In addition, the surface oxidation behavior of the HEA is probed using X-ray photoelectron spectroscopy (XPS) and is likewise found to be consistent with our theoretical findings.

## Methods

### Alloy Preparation

Equimolar amounts of pure (99.99+%) Co, Cr, Fe, and Ni powders were arc-melted in an argon atmosphere on a water-cooled copper hearth with a tungsten electrode. The polycrystalline button of CoCrFeNi thus formed was turned over several times and re-melted to ensure a homogeneous sample. Thereafter, the button was sliced into thin wafers (ca. 8 × 5 × 1 mm^3^) by electrical discharge machining, and each wafer was mechanically polished with alumina powder (0.2 μm, 99.99%, Alfa Aesar).

### Sample Characterization

X-ray diffraction (XRD) measurements were performed using a PANalytical Empyrean X-ray diffractometer employing a reflection transmission spinner with a minimum step size of 0.026° through a range of 10°–90°. Moreover, electron-backscattered diffraction (EBSD) and energy-dispersive X-ray spectroscopy (EDS) measurements were performed using a FEI Quanta 3D DualBeam FEG FIB-SEM at the Shared Instrumentation Facility at Louisiana State University.

X-ray absorption and photoelectron experiments were performed at LSU’s synchrotron facility, Center for Advanced Microstructures and Devices (CAMD). Extended X-ray absorption fine structure (EXAFS) spectra were collected for the metal K edges in the fluorescence mode at a multipole wiggler beam line in the range of −200 to +800 eV relative to their respective metal foil energy. For comparison, EXAFS spectra were also collected for the standard foils (Cr 5.989 keV; Fe 7.112 keV; Co 7.709 keV; and Ni 8.333 keV) and the HEA at the same beam line in the transmission mode. The intensity was corrected with Booth fluorescence correction in Athena as the composition is well known.^27^ EXAFS spectra were collected over as wide an energy range as possible because all the four 3d elements are close to one another on the periodic table. The above experiments were performed on as-cut and mechanically polished wafers.

XPS experiments were performed in an UHV chamber that was equipped with an Omicron DAR 400 X-ray source and an Omicron EA125 hemispherical electron energy analyzer. All XPS spectra were taken using Mg Kα X-rays (*h̵*ω = 1254 eV) with an analyzer pass energy of 25 eV. Before performing XPS experiments on a wafer, it was inserted into a ultra-high vacuum (UHV) chamber with a base pressure of 1 × 10^–10^ Torr and cleaned by repeated cycles of neon ion sputtering (45 min), heating (to 800 °C in ca. 5 min), and cooling (to ambient temperature over a period of ca. 30 min). Sample cleanliness was checked with XPS to verify the absence (<2%) of oxygen and carbon in the near-surface (<1 nm) region in the spectra. In addition, angle-dependent XPS was also performed with a Specs Phoibos 150 Electron Analyzer with a monochromated Al Kα X-ray (*h̵*ω = 1487 eV) source. Dosing of oxygen (99.999%), manually controlled via a leak valve, was typically done at a chamber pressure of ca. 10^–5^ Torr for a pre-set time (1 L = 10^–6^ Torr·s) at ambient temperature.

### Electrochemical Measurements

Polarization curves were measured using linear sweep voltammetry at a scan rate of 20 mV/s from 0 to −0.7 V with a SP-150 potentiostat (BioLogic) in a 0.5 M H_2_SO_4_ aqueous solution (ACS grade; pH 0.3) at ambient temperature and pressure. Graphite was used as the counter electrode, and Ag/AgCl (BASi-5B, with 3 M NaCl) was used as the reference electrode. The electrolyte solution (50 mL) and graphite were freshly prepared in each experiment to prevent ion contamination. The working electrode was a slice of the HEA that was 0.5 mm in thickness and 5.6 mm in diameter, cut and polished using the procedure described above, and attached to the electrical wire with a silver paste for electric conductivity. The electrode was sealed with a crystal bond (509) to ensure a constant exposed active surface area. Resistance was corrected in all electrochemical experiments by performing resistance check before any waveform, and it was found to be negligible before potentiostat correction. Besides the HEA, Cr foil (99.997%, Alfa Aesar), Co rod (99.995%, Alfa Aesar), Fe foil (99.99%, MTI Corp.), Ni foil (99.99%, MTI Corp.), SAE 304 stainless steel (referred to below as SS304), and a Pt electrode were also tested using the same setup. SS304 was included for comparison because it is a common alloy of Fe, Cr, and Ni (17–20% Cr, 8–11% Ni, and a few other elements at 2% or less, with the balance being Fe) and is known for its resistance to a variety of corrosive media. The HEA and Pt electrodes were mechanically polished with alumina powder to refresh the surface and to limit oxidation before each test. The Co, Cr, Fe, Ni, and SS304 electrodes were prepared from fresh metal foils for respective tests. Currents were normalized by the geometric area of each electrode.

The electrochemical stability of the HEA was evaluated by cycling between 0 and −0.45 V in a 0.5 M H_2_SO_4_ at a rate of 20 mV/s for 1000 times (a total of 12.5 h). For comparison, another sample was separately immersed in 0.5 M H_2_SO_4_ for the same amount of time without an applied potential. Both the post-reaction electrolyte solution and the control electrolyte solution were diluted with 5% HNO_3_ and analyzed for Co, Cr, Ni, and Fe content with an Optima 8000 inductively coupled plasma optical emission spectrometer (ICP-OES, PerkinElmer).

### Computational Methods

Density functional theory (DFT) calculations were performed in the generalized gradient approximation using the Perdew, Burke, and Ernzerhof functional (GGA-PBE).^28^ The properties of the bulk CoCrFeNi HEA, which crystalizes in an A1 face-centered cubic (fcc) structure,^29^ were calculated using two methods: (1) the Korringa–Kohn–Rostoker coherent potential approximation (KKR-CPA), which requires only one-atom unit cells for homogeneous fcc alloys, and (2) the plane-wave pseudo-potential method as implemented in the Vienna Ab Initio Simulation Package (VASP),^30^ which involves the use of large, explicit unit cells with an equimolar composition (see below). Calculations for surface adsorption were done using VASP on explicit slab models cut from the large bulk supercell (see below).

The KKR method is an all-electron Green’s function method for calculating the electronic structure and total energy.^31−34^ The CPA is a mean-field theory method for calculating the configurationally averaged Green’s function for substitutionally disordered alloys.^33−36^ Together, the KKR-CPA performs configurational averaging and DFT charge self-consistency simultaneously. The atomic potentials were modeled within the atomic sphere approximation,^37^ supplemented by a variationally optimized potential energy zero, resulting in formation energies matching full potential results.^38^ The valence states were expanded in a spherical harmonic basis that included angular momentum up to *l*_max_ = 5 (i.e., electron symmetries of s, p, d, and f electrons). Green’s function (and hence site-decomposed electronic densities) for the system was found using a semi-circular Gauss–Chebyshev contour integration with 20 complex energy points. As it approaches singularity when the energies approach the Fermi level (ε_F_), two Monkhorst–Pack (MP) *k*-point meshes^39^ were used. For energies with an imaginary part greater than 0.25 Ry, a 12 × 12 × 12 MP *k*-point mesh was used, and for energy points with an imaginary part less than 0.25 Ry (i.e., in the vicinity of ε_F_), a denser 18 × 18 × 18 MP *k*-point mesh was used. These parameters were determined to provide an accuracy of a 10^–5^ Ry in the total energy.

**Figure 1:**
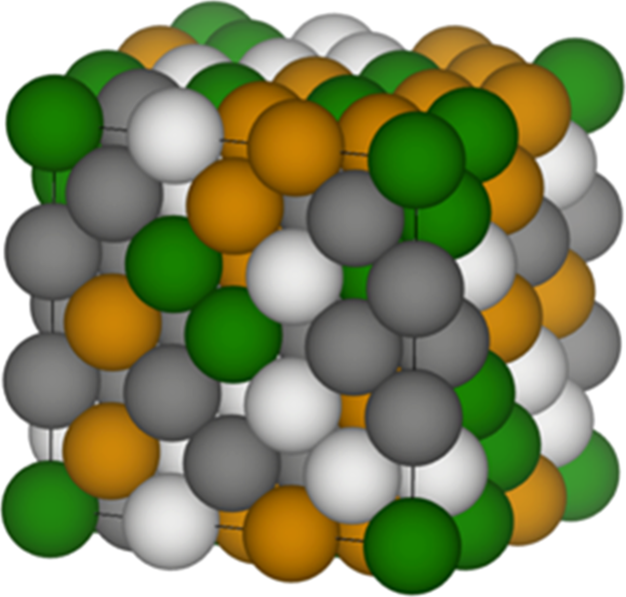
108-atom SCRAPs supercell for CoCrFeNi HEA (Co = green; Cr = dark gray; Fe = yellow; and Ni = white).

The large bulk supercell was generated using the Super-Cell Random Approximates (SCRAPs) method by Johnson and co-workers^24^ and calculated using VASP. While no supercell of a finite, practical size could be a unique representation of the HEA, the 108-atom Co_27_Cr_27_Fe_27_Ni_27_ supercell was generated using a hybrid Cuckoo Search algorithm^40^ with combinatorially optimized atomic point probabilities (site compositions) and pair probabilities (related to atomic short-range order) to ensure a configuration with zero short-range order for up to three nearest-neighbor shells at every atomic site (Figure 1), mimicking a “scrap” of the actual equimolar homogeneous disordered alloy.

**Figure 2:**
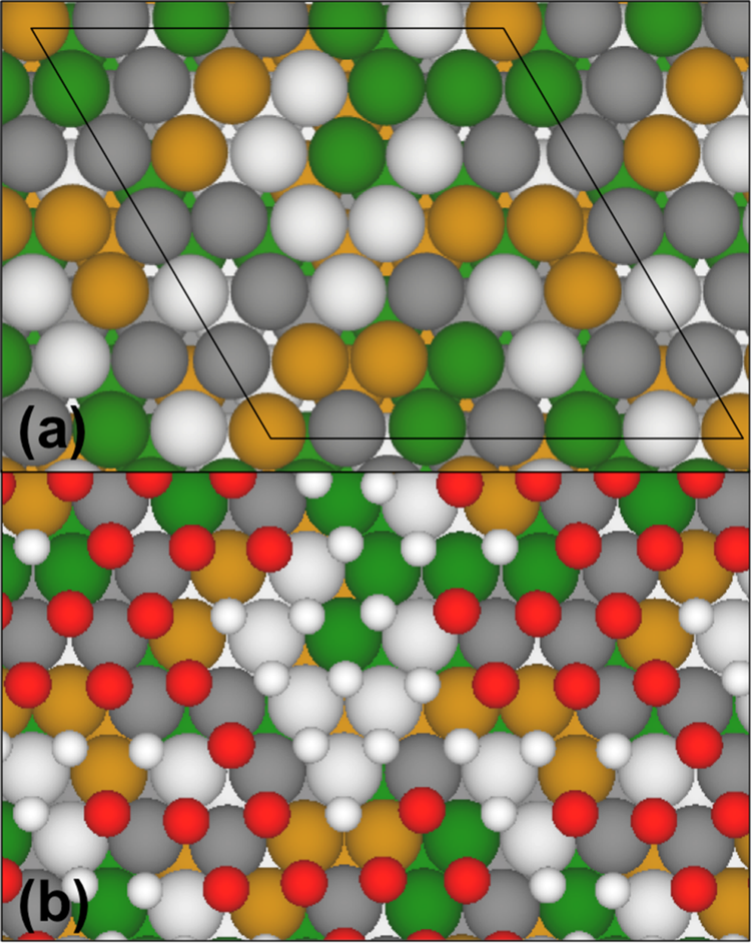
(6 × 6) surface model for the (111) facet of the CoCrFeNi HEA. (a) Clean surface with the surface unit cell outlined (black line); (b) extent to which the surface will be oxidized at 0 V vs SHE and pH 0, with all remaining fcc sites occupied by H atoms. Color code: Co = green; Cr = dark gray; Fe = yellow; Ni = white; O = red; and H = white (small).

Calculations for surface adsorption on the CoCrFeNi HEA were done using VASP on slab models cut from the large SCRAPs bulk supercell. The potentials due to the core electrons were described using the projector augmented wave method.^41^ The Kohn–Sham one-electron valence states [Cr(3p3d4s), Fe(3d4s), Co(3d4s), Ni(3d4s), O(2s2p), and H(1s)] were expanded in a plane-wave basis up to 650 eV. The (111) slab model had a (6 × 6) surface unit cell and consisted of four layers of metal (Figure 2a). The (100) slab model had a (4√2 × 4√2) surface unit cell and also consisted of four layers of metal (Figure S1 in the Supporting Information). See the Supporting Information also for the atomic coordinates of the as-cut slab models. The (111) facet is the lowest energy facet of pure fcc metals. For the HEA, we calculate the average surface energy of the two as-cut, unrelaxed slab models to be +0.91 and +1.72 eV/atom, respectively, for (111) and (100), suggesting (111) also to be the lowest energy facet of the HEA. Periodic slabs were separated in the *z*-direction by five layers equivalent of vacuum, with electrostatic decoupling applied in the *z*-direction.^42^ The surface Brillouin zone was sampled on a 2 × 2 × 1 MP *k*-point mesh for each surface unit cell. Increasing the density of the *k*-point mesh to 3 × 3 × 1 was found to affect the total energy of the system by less than 0.1 eV. A first-order Methfessel–Paxton scheme was used to smear the electronic states with a width of 0.2.^43^ All total energies were extrapolated to 0 K.

Adsorption was modeled on one of the two sides of each slab only. All adsorbates and the two metal layers nearest to the adsorbates in the slab were relaxed, while the remaining two layers were held fixed at their bulk positions. Geometry optimization was considered to be converged when the residual force in each relaxed degree of freedom in the system was lower than 0.03 eV/Å. The average adsorption energy (Δ*Ẽ*_A*_) of *n* H or O atoms was calculated as , where *E*_*n*A*_, *E*_surf_, and *E*_A_2_(g)_ are the total energies of a surface plus *n* adsorbate atoms, the surface without the adsorbates, and the reference gas-phase species, which is H_2_ or O_2_ for the adsorption of atomic H or O, respectively.

The free-energy correction to the adsorption energy of an adsorbed H or O atom was calculated as
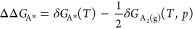
1

See ref (44) for how the free energy corrections (δ*G*) to the total energies are defined for adsorbed atomic H and O (δ*G*_A*_(*T*)) and for gas-phase molecular H_2_ (δ*G*_H_2_(g)_(*T*,*p*)). δ*G*_O_2_(g)_(*T*,*p*) was calculated using the approach of Martínez et al. to avoid the error inherent in the GGA total energy of gas-phase O_2_.^45^ At ambient temperature and pressure (298.15 K and 1 bar), ΔΔ*G* was estimated to be 0.21 eV (in agreement with Nørskov et al.^46^) and 0.13 eV per adsorbed H and O atom, respectively. The effect of an aqueous phase on Δ*Ẽ*_H*_ was tested using the implicit solvation model of Mathew et al.^47^ for all the monometallic surfaces listed in Table 1 and found to be no larger than 0.01 eV per H atom in any of the cases. Therefore, it was neglected from the HEA surface calculations.

**Table 1:** Average Total Energy, Average Free Energy, and Differential Free Energy of Adsorption for Atomic H (Δ*Ẽ*_H*_, Δ*G̃*_H*_, and d*G*_H*_, in eV per H Atom) on Respective Surfaces^a^

Surface	Δ*Ẽ*_H*_	Δ*G̃*_H*_	d*G*_H*_
Pt(111)	–0.38	–0.17	–0.13
Ni(111)	–0.55	–0.34	–0.33
Co(0001)-1O	–0.11	+0.10	–0.03
Fe(110)-2O	+0.01	+0.22	+0.27
Cr(110)-3O	–0.22	–0.01	–0.01
HEA(111)			
top side	–0.68	–0.47	–0.40 ± 0.08
top side-18O	–0.33	–0.12	–0.09 ± 0.10
reverse side	–0.66	–0.45	–0.39 ± 0.06
reverse side-15O	–0.37	–0.16	–0.13 ± 0.09
HEA(100)	–0.62	–0.41	–0.38 ± 0.05
HEA(100)-14O	–0.36	–0.15	–0.09 ± 0.13

a Values are based on full H coverage, that is, with H atoms occupying all open fcc sites. Partially oxidized surfaces are labeled by the number of residual O atoms per surface unit cell. All pure metals are calculated on (2 × 2) surface unit cells. Δ*Ẽ*_H*_ on Pt(111) and Ni(111) are taken from ref (23). Co(0001), Fe(110), and Cr(110) are calculated using the same parameters as Pt(111) and Ni(111). The average and standard deviation of d*G*_H*_ are calculated by individually sampling 18 H atoms on the HEA(111) (Figure 2) and HEA(100) (Figure S1) models and 21 H atoms on the reverse side of the HEA(111) model (Figure S2).

## Results and Discussion

### Theoretical Modeling

CoCrFeNi crystalizes in an A1 fcc structure in a paramagnetic (PM) state with a lattice constant of 3.564 Å.^48,49^ It is larger than what Vegard’s law would predict based on an elemental nearest-neighbor, equimolar assumption and is larger than pure Ni (3.52 Å). KKR-CPA determines the equilibrium lattice constant of the HEA to be 3.529 Å in the PM state, with the average *magnitude* of the magnetic moments of the component elements (Co, Cr, Fe, and Ni) being 0.016, 0, 1.850, and 0 μB, respectively. According to KKR-CPA, the electronic ground state is actually ferromagnetic (FM), with a slightly larger lattice constant of 3.553 Å and average moments of 1.218, −0.953, 2.152, and 0.313 μB, respectively. Energetically, the FM state is 21.5 meV/atom more stable than the PM state. Assuming that the transition of the magnetic state occurs at a temperature (i.e., the Curie temperature) where interatomic diffusion does not occur at appreciable rates, this difference in total energy can be used to estimate *T*_C_ based on a mean-field Heisenberg-like model^50^ to be as low as *T*_C_ = 2/3 (21.5 meV)/*k*_B_ = 166 K,^51^ which may be why it has not been readily detected experimentally.

In handling for electrochemical applications, it is difficult to avoid exposure of electrode materials to oxygen-containing molecules in the environment, for example, O_2_ and water. To explore the potential catalytic properties of the HEA, surface oxidation needs to be taken into account. At ambient conditions, we do not expect the surface of the HEA to undergo significant reconstruction, which justifies the use of a fcc(111) slab as a model for the HEA surface. Just as no bulk supercell of any practical size can fully represent the bulk HEA, no finite-size surface model can uniquely represent the HEA surface. Nonetheless, we use approximate surface models to capture potential surface heterogeneity and coverage effects. For practicality, we use a (6 × 6) surface unit cell for the (111) facet and a (4√2 × 4√2) surface unit cell for the (100) facet constructed as described in the Methods sections with the calculated lattice constant of the PM state, 3.529 Å, to model oxygen and hydrogen adsorption using plane wave-based GGA-PBE calculations. All VASP calculations have been done without spin polarization as an approximation of the PM state because the prohibitive costs of non-collinear calculations and exhaustive enumeration of possible initial magnitudes and directions of magnetic moments on the metal atoms needed to model PM states.

**Figure 3:**
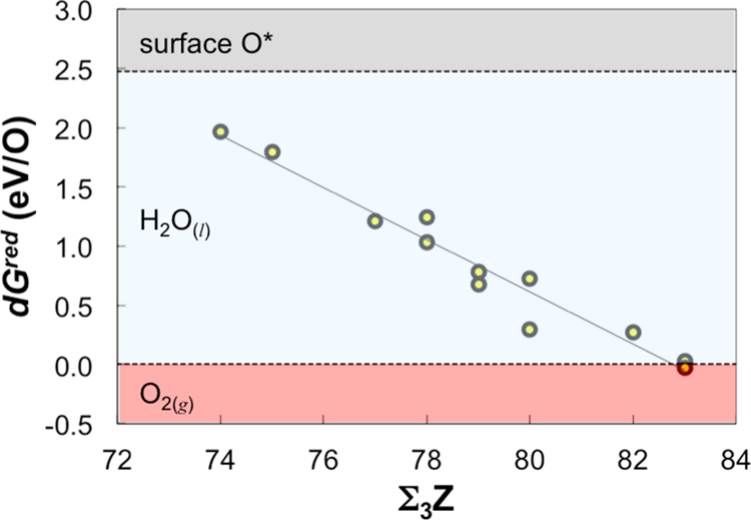
Differential reduction free energy (d*G*^red^) of adsorbed atomic oxygen at 1 ML coverage on a randomly chosen subset of the fcc sites on the HEA surface model (Figure 2a) vs the sum of atomic numbers of the three metal atoms that make up a site (Σ_3_*Z*). This particular surface model does not exhibit any pure Cr (Σ_3_*Z* = 72) or pure Ni (Σ_3_*Z* = 84) fcc sites. The three shaded regions represent different most stable phases of O with respect to d*G*_red_: adsorbed O atom (>2.47 eV); liquid water (referenced to gas-phase O_2_ and H_2_ at the standard state; < 2.47 eV); and gas-phase O_2_ (<0 eV).

We limit atomic adsorptions to fcc 3-fold hollow sites on the (111) surface, shown in Figure 2a, which are the preferred adsorption sites by atomic hydrogen and oxygen on the (111) facets of fcc metals.^52−54^ O_2_ dissociation on base metals is known to have small barriers.^54,55^ We assume that upon exposure to O_2_, the HEA surface is rapidly covered in dissociated atomic oxygen. To determine the extent of oxygen coverage on the surface when subject to ambient O_2_, we start with 1 ML of adsorbed O atoms (on all the fcc sites on the (6 × 6) surface) and calculate the differential reduction energy (d*G*^red^) of an fcc site as follows
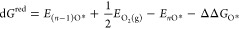
2where *n* = 36. This is the energy required to remove an adsorbed O atom from a given site at overall 1 ML coverage and put it in gas phase as part of an O_2_ molecule. A negative d*G*^red^ indicates that it is energetically favorable for a site to remain un-oxidized. The results, when plotted against the sum of the atomic numbers of the three metal atoms that immediately make up a given fcc site, show a linear trend (d*G*^red^ = −0.22(Σ_3_*Z*) + 18.24, *R*^2^ = 0.96; Figure 3), indicating that the elemental makeup of the first nearest-neighbor shell has a strong effect on the site reducibility. According to the trend line, d*G*^red^ drops below the free energy of gas-phase O_2_ when Σ_3_*Z* exceeds 83. That is, all sites except those composed of all Ni atoms are predicted to be oxidized upon exposure to ambient O_2_, resulting in a nearly complete oxygen coverage. Note that the trend line would shift upward and its slope may change if the coverage of atomic oxygen decreases due to reduction processes. The linear correlation does not imply that the validity of the Σ_3_*Z* ansatz extends beyond the given HEA.

When an HEA(111) surface that has been exposed to ambient O_2_ is then exposed to an acidic solution under a potential, some of the O atoms would be removed as H_2_O via reduction by H^+^/e^–^. To compare the stability of adsorbed O atoms to liquid water instead of gas-phase O_2_, we note that
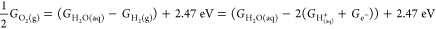
3at standard conditions, which replaces the O_2(g)_ term in eq 2. Here, the free energy of aqueous-phase H_2_O is taken to be equal to that of gas-phase H_2_O at 0.0317 bar, that is, the saturation pressure of H_2_O at ambient temperature. In an electrochemical setting, gas-phase H_2_ at standard conditions is equivalent to protons in an aqueous solution at a concentration of 1 M (i.e., pH 0) and e^–^ at 0 V versus the standard hydrogen electrode (SHE). A typical electrolyte solution used for testing HER activity is a dilute aqueous solution of sulfuric acid,^11,21,56−59^ which has a pH slightly higher than 0. Thus, the reduction limit for an adsorbed O atom with respect to liquid water at 0 V and pH = 0 lies at 2.47 eV positive of the reduction limit with respect to gas-phase O_2_. The stability limits are indicated in Figure 3.

Based on this understanding, we take the 1 ML O-covered HEA(111) surface and sequentially remove the O atoms in the order of largest to smallest Σ_3_*Z* values and stop when d*G*^red^ for removing the next O atom would exceed the liquid water level. This procedure allows us to construct a partially oxidized HEA surface where 19 of the 36 fcc sites are oxidized. The remaining 18 open fcc sites (or 50% of all the fcc sites), which are predominantly composed of Ni and Co, are then populated with H atoms (Figure 2b). We have also considered the O atoms as adsorption sites for H but found them to be considerably less stable than the open fcc sites for H. In comparison, the same procedure when applied to the pure metals predicts that Pt(111) and Ni(111) should remain in a clean (i.e., un-oxidized) state, whereas some O atoms remain on Co(0001), Fe(110), and Cr(110). Note that formation of bulk oxide surfaces is not considered for the pure metals.

The free energy of adsorption for atomic H (Δ*G*_H*_) has long been used as the activity descriptor for HER for metals.^46,60,61^ We calculate the average Δ*G*_H*_, or Δ*G̃*_H*_, for adsorbed H atoms at full coverage^61^ according to eq 4
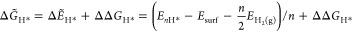
4

For the pure metal surfaces, *E*_surf_ is the total energy of a clean surface and *n* = 4 for (2 × 2) surface unit cells. For the un-oxidized HEA(111) surface, *n* = 36 (Figure 2a). For the partially oxidized HEA(111) surface (Figure 2b), *E*_surf_ is the energy of the HEA surface with 18 O atoms and *n* = 18. Furthermore, the corresponding differential free energy of adsorption (d*G*_H*_) is also calculated according to eq 5
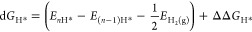
5Here, *E*_(*n*–1)H*_ is the total energy of a fully H-covered surface with one H atom removed. The results for the HEA are listed in Table 1 in comparison to Pt(111), Ni(111), Co(0001), Fe(110), and Cr(110).

The un-oxidized HEA(111) surface overbinds H compared to Ni(111), which in turn overbinds H compared to Pt(111), indicating that they are both less active than Pt. On the other hand, the reactivity toward H on the open sites of the partially oxidized HEA surface is closer to that on Pt(111) than both the un-oxidized Ni(111) and partially oxidized Cr, Fe, and Co surfaces, the latter adsorbing H too weakly, whether on the basis of Δ*G̃*_H*_ or d*G*_H*_. From this, we conclude that the HEA surfaces should be more active for HER than all the component metals. It remains debated whether base metals such as Ni are less active than Pt because they overbind or underbind H.^46,62^ Our approach suggests that the adsorbed O atoms play an important role in moderating the surface reactivity of the metal surfaces toward H in acidic electrolytes. This is different from the role played by surface oxide species in promoting water dissociation in HER in alkaline solutions.^63^

As indicated in Table 1, the differential binding strength of individual H atoms at high coverage varies tightly around a mean value. Approximately 55–70% of all the open sites on the partially oxidized HEA surfaces are within ca. 0.1 eV of the Pt(111) value, assuming normal distribution. Fewer than 4% of the sites on the partially oxidized HEA surfaces that we have examined exist outside two respective standard deviations. Although on pure metals all surface sites of a given symmetry are identical before any coverage effect sets in, it is not true on the HEA surfaces. Because of this, we could expect responses to macroscopic catalytic activity measurements, whether taken in a reactor or in an electrochemical cell, to be intrinsically less sharp than on pure metals. Incidentally, d*G*^red^ in Figure 3 yields +0.83 ± 0.64 eV for atomic O at 1 ML, a significantly wider spread than for atomic H. A flatter potential energy surface for H adsorption is commonly seen on transition metal surfaces than for O adsorption.

If the electrode potential is swept from 0 V to more negative values, the reduction power of H^+^/e^–^ would increase and more of the O atoms would be removed. Due to the stronger binding of atomic O than atomic H at medium to low coverage, when any provided overpotential removes additional O atoms from the surface, the newly opened sites become immediately available for adsorption of H atoms and active for HER. For example, we estimate by the same procedure mentioned above that 50% more fcc sites will become reduced at an overpotential of 0.4 V than at 0 V. 0.4 V × *e*^–^ = 0.4 eV exceeds the amount by which Δ*G̃*_H*_ varies from the partially oxidized state to the un-oxidized state of the HEA surface (spanning ca. 0.3 eV, Table 1).

The same procedure is repeated on the reverse side of the (6 × 6) (111) slab, yielding another representation of CoCrFeNi(111) (Figure S2 in the Supporting Information), and on one side of the (4√2 × 4√2) (100) slab (Figure S1 in the Supporting Information). For the reverse side of the (6 × 6) (111) slab, the partially oxidized surface that results from the same procedure has 21 open fcc sites that are populated with H atoms. The Δ*G̃*_H*_ for the 21 H atoms is 0.02 eV weaker than on Pt(111), whereas on the first model it is 0.05 eV weaker than on Pt(111) (Table 1). For the (4√2 × 4√2) (100) slab, the resulting partially oxidized surface has 18 open 4-fold hollow sites that are populated with H atoms (bridge and top sites are markedly less stable than hollow sites). The Δ*G̃*_H*_ for the 18 H atoms is 0.03 eV weaker than on Pt(111) (Table 1). Thus, in their un-oxidized state, all of these HEA surface models overbind H compared to Ni(111), while their reactivity toward H adsorption is much closer to Pt(111) in the partially oxidized state.

### Alloy Synthesis

**Figure 4:**
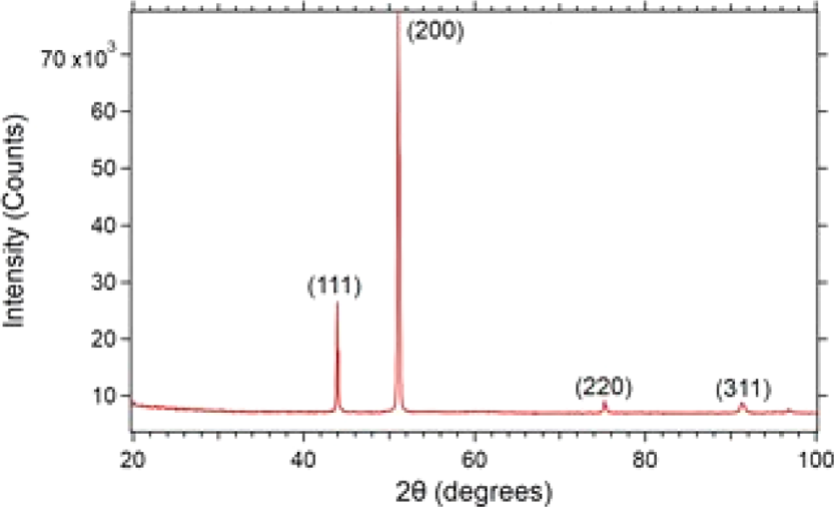
XRD of a typical fcc CoCrFeNi sample.

**Figure 5:**
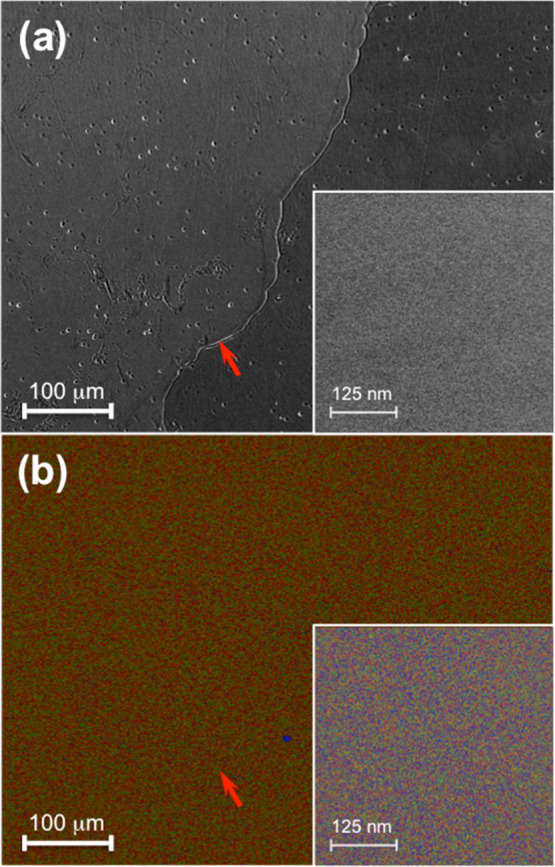
(a) SEM and (b) corresponding EDS images of a typical CoCrFeNi HEA sample showing the same area containing a grain boundary (indicated by red arrows). Insets show an enlarged area of the same sample away from the grain boundary.

To test the theoretical predictions, we synthesize CoCrFeNi via arc-melting of the pure metals in argon. Figure 4 shows the XRD of a typical freshly made and cut CoCrFeNi sample. The strong 2θ peaks, with a narrow line width at 44°, 51°, 75°, and 91° correspond to diffraction from the (111), (200), (220), and (311) planes, respectively, of a homogeneous, single-phase fcc crystal structure with large, coherent domains. The lattice constant is determined to be 3.56 Å in agreement with the literature,^48,49^ resulting in a nearest-neighbor distance of 2.52 Å. Alloying Cr, Fe, Co, and Ni produces little lattice distortion because the four elements are neighboring 3d transition metals on the periodic table and have nearly identical interatomic distances (ca. ±0.5%). EDS and scanning electron microscopy (SEM) (Figure 5) show no compositional inhomogeneity down to 10 nm. The overall Co:Cr:Fe:Ni composition is measured to be 26.5:23.7:25.0:24.8, very close to equimolar composition. EDS also shows that the near-surface composition (<80 nm) does not vary along grain boundaries, so we do not expect grain boundary effects to be a significant factor. Additionally, EBSD data (Figure S3 in the Supporting Information) confirm that we have obtained large grain structures 100–1,000 μm in size.

**Figure 6:**
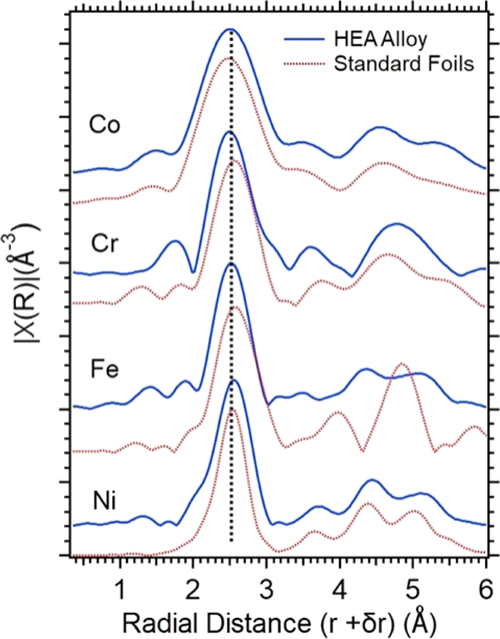
χ(*R*), the Fourier transforms of EXAFS oscillations, showing the radial distance for the nearest-neighbor shells. The dashed line indicates a radial distance of 2.52 Å.

Fourier transforms of EXAFS spectra yield the radial distribution of the coordination shells for each of the four elements in the HEA alloy (solid blue, Figure 6) versus that of pure metallic foils (dashed red, Figure 6). To compensate for phase shifts, the spectra of the HEA and the foils are normalized by a linear shift of the same amount, so that the average center of the nearest-neighbor peaks of the four components in the HEA corresponds to the XRD result of 2.52 Å. The EXAFS analysis indicates that the four elements have nearly identical first nearest-neighbor distances (the positions of the largest peaks) compared to the foils, with some subtle differences in the second and subsequent neighbor distances. Beyond 3 Å, while the spectra of Co and Ni in the HEA (blue) and in the pure metal foils (red) are nearly identical, clear differences exist between the HEA and the Cr and Fe foils because pure Cr and Fe prefer the bcc structure.

### Electrochemical Testing

**Figure 7:**
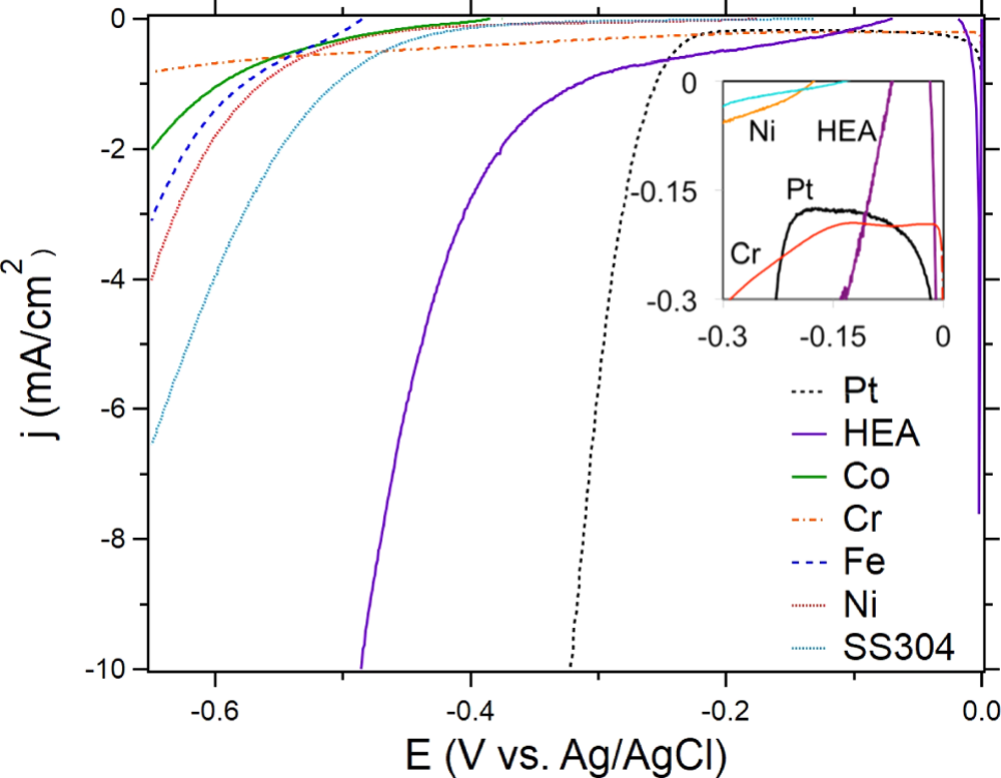
Polarization curves of CoCrFeNi, individual base metals, SS304, and Pt in 0.5 M H_2_SO_4_ at ambient temperature and pressure at a scan rate of 20 mV/s. The inset shows a magnified view of the area around the origin. See Figure S4 in the Supporting Information for the corresponding Tafel plot.

The electrochemical HER activity of the HEA is then tested in 0.5 M H_2_SO_4_, as well as the activities of the individual component metals, SS304, and Pt for comparison. As shown in Figure 7, Pt is the most active HER electrode among these materials. The onset potential (*U*) for HER as measured at a current density (*j*) of 1 mA/cm^2^ is found to be −0.26 V (Pt), −0.32 V (HEA), −0.51 V (SS304), and −0.57 V (Ni). Based on *U*, the HEA is only 60 mV less active than Pt, which is comparable to the HER activity of MoS_2_ in H_2_SO_4_ aqueous solutions,^56−58^ and is more active than each of the base metals by 250 mV and more. These results thus validate our theoretical predictions. Among the base metals, Ni is the most active, whereas Cr remains mostly passivated between 0 and −0.7 V and shows minimal electrochemical activity.^64^ At *j* = 1 mA/cm^2^, Ni has an overpotential of 310 mV compared to Pt, which is consistent with the electrochemical studies of Ni in acidic solutions reported in the literature.^21,59,65,66^ For SS304, *U* is 250 mV lower than Pt, which is also in line with the previously reported electrochemical activity for stainless steel in acidic solutions.^67,68^ The current feature observed for Pt at 0 V is due to sulfate anion adsorption.^69,70^ It is unclear whether the origin of the 0 V feature for the HEA is also due to sulfate adsorption or to reduction of certain surface oxide species.^64,67^

**Figure 8:**
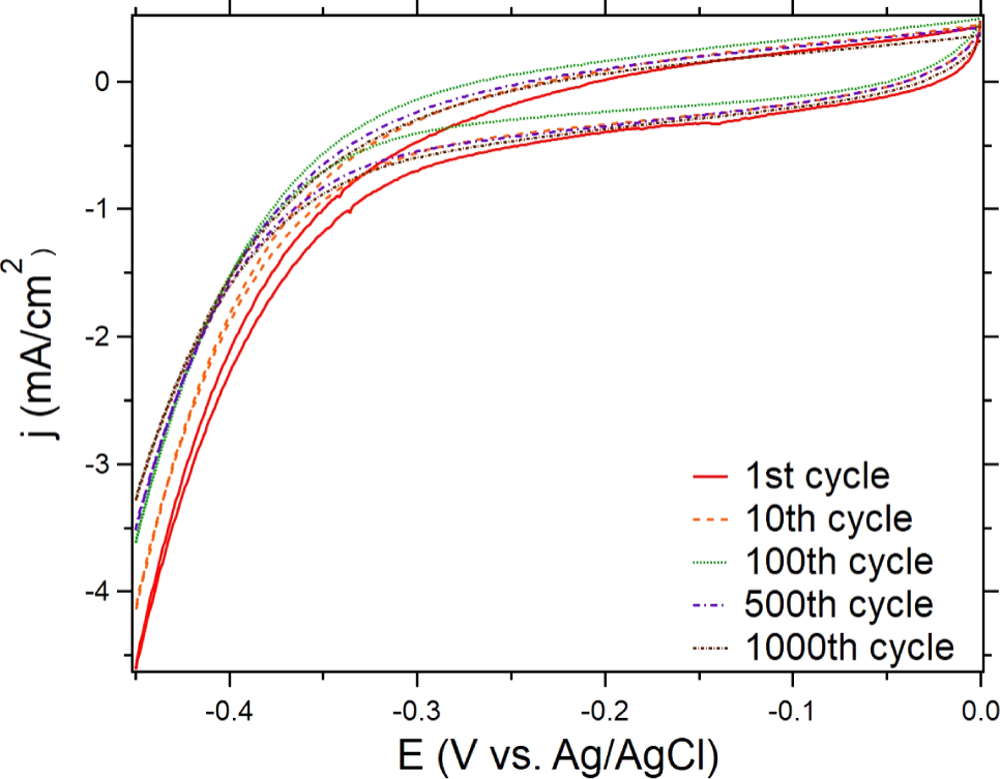
Cycling of CoCrFeNi between 0 and −0.45 V vs Ag/AgCl in a 0.5 M H_2_SO_4_ aqueous solution at a scan rate of 20 mV/s at ambient conditions.

The stability of CoCrFeNi is tested by cycling between 0 and −0.45 V at a rate of 20 mV/s for 1000 times (for a total of 12.5 h). The results are shown in Figure 8. There is some current degradation during first 100 cycles, but the current stabilizes after this stage. Dissolved metal ions in the electrolyte solution have been examined with ICP-OES (Table 2). In the control experiment where no voltage is applied, small amounts of all the component metals are dissolved and detected in the electrolyte solution. The amounts of dissolved metal ions increases by 5–10 fold in the cycling test, but the total weight loss amounts to only ca. 0.1 wt % of the starting HEA electrode. The differences do not suggest a preferential loss of any particular metal. The occupation of surface defect sites, where dissolution of metals likely commences, by Cr or the other oxide species that are more resistant to dissolution than the metals may have protected the metallic domains of the HEA from rapid dissolution.

**Table 2:** Amounts (in μg) of Dissolved Metals from CoCrFeNi Immersed in 0.5 M H_2_SO_4_ at an Open Circuit and after 1000 Cycles between 0 and −0.45 V, Both for 12.5 h

treatment	Co	Cr	Fe	Ni
fresh electrolyte solution	3.27	1.92	0.00	2.76
open circuit	3.60	2.64	1.62	3.57
1000 cycles	21.51	18.12	19.62	22.32

Superior corrosion resistance properties of HEAs including CoCrFeNi have been noted before.^71^ In 0.5 M H_2_SO_4_, Ni alloys and stainless steel are less corrosion resistant than CoCrFeNi.^67,72^ While the HEA is by no means free of dissolution, there is no indication that the button electrode suffered any significant loss of electrochemical active surface area as often occurs with carbon-supported nanoparticles used as active materials.

### Oxidation Characteristics

**Figure 9:**
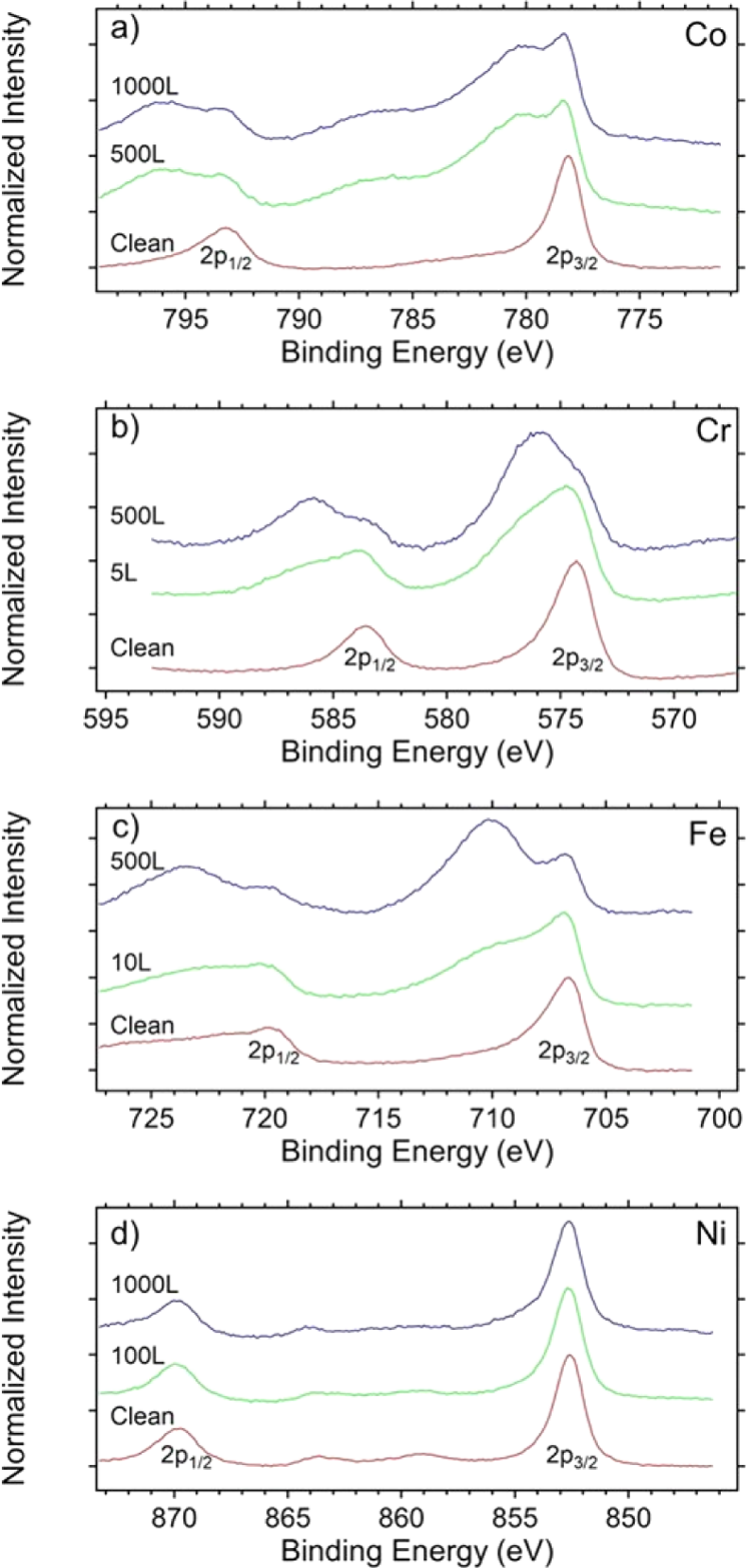
XPS spectra of the 2p peaks of the four elements in the HEA: (a) Co, (b) Cr, (c) Fe, and (d) Ni at various dosages (1 L = 1 × 10^–6^ Torr·s) of O_2_ at ambient temperature.

To begin exploring the oxidation characteristics of the HEA, we expose a clean sample to increasing doses (5, 10, 100, 500, and 1000 L) of O_2_ in UHV. XPS spectra are taken after each dose to monitor how the surface electronic structure evolves. While our current UHV experiments do not probe surface oxidation of the HEA in electrochemical settings, they serve as a useful reference for future, more in-depth studies of this alloy, and more immediately, they help validate our theoretical methodology by verifying whether surface Ni is indeed less likely to be oxidized than the other elements. Figure 9 shows the selected spectra of each element to represent the onset and saturation of oxidation of that element versus a clean sample. The spectra of a clean surface exhibit 2p_1/2_ and 2p_3/2_ levels characteristic of the individual metals.^73^ With exposure to O_2_, additional features appear at higher binding energies, indicating the formation of oxides.^73^ Cr oxides form quickly (within 5 L O_2_) and saturate well below 500 L O_2_. Fe and Co are the next to oxidize, saturating at around 500 L O_2_. Because the peaks due to many related oxide species (e.g. CrO_2_ and Cr_2_O_3_) overlap in energy, the determination of the relative concentration of each oxide species is beyond this study. Ni does not show any new peak associated with nickel oxides up to 1000 L O_2_, and as can be seen in Figure 9, there is no shift in the position of the 2p_3/2_ peak of Ni even at 1000 L O_2_, which suggest lack of Ni oxide formation consistent with our calculations. Moreover, there is no discernible indication of a characteristic NiO shake-up at a binding energy of 861 eV,^74^ while the metallic shake-up persists at 858 eV. In comparison, Ni oxides form on pure Ni at much lower doses of O_2_.^75^

Furthermore, XPS spectra are taken by varying the electron emission angle (i.e., the take-off angle) between the surface normal of the sample and the detector of 0°, 20°, 40°, and 60° for a clean sample and with oxygen exposures. As the emission angle is adjusted to a more grazing configuration, the probe depth includes a higher contribution from the surface passivation layer. This method, which is a standard XPS technique, is based on the universal curve of electron escape depths and their relation to the emission angle.^76^ By comparing each metal oxide/metal ratio, this technique provides information on how the individual^76^ metals and their oxides contribute to this layer. For each of the clean surface spectra, the area attributing to the 2p_3/2_ metal peak was fit using the commercial software CasaXPS^77^ following the removal of the secondary electron background using the standard Shirley method.^78^ For the data taken following O_2_ exposure, the spectra were fit with the same parameters as those obtained from the peak fitting of the clean samples. Additional peaks were then fit to the spectra of the constituent metals to represent the contribution of the corresponding oxides to the spectra.

**Figure 10:**
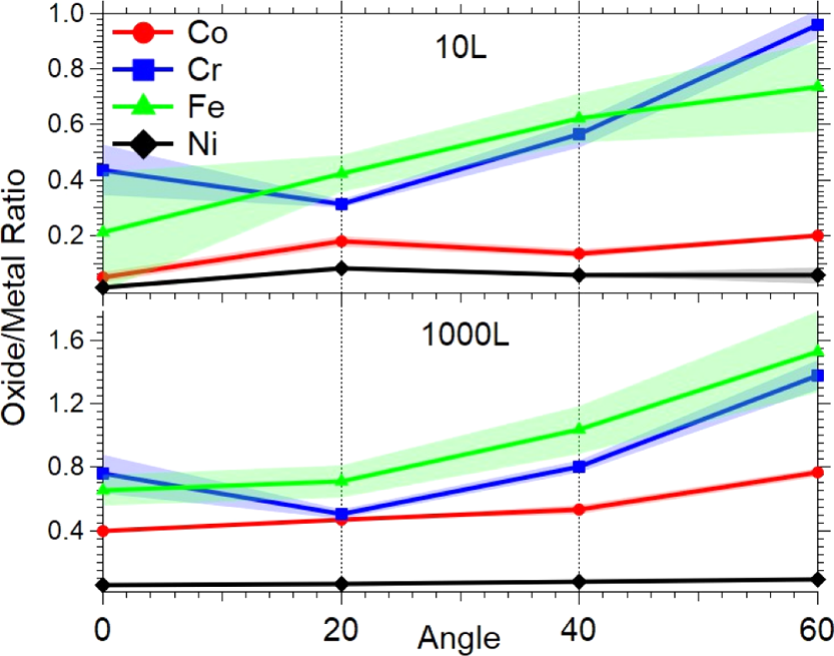
Ratio of oxide to metal peak areas at 10 (top) and 1000 (bottom) L of O_2_ at various photoemission angles. Shades represent errors associated with the data.

Figure 10 shows a plot of the ratio of the oxide to metal peak areas obtained by this procedure at two dosages (10 and 1000 L O_2_) as a function of emission angle. It can be seen that the contribution to the spectra from the oxide features increases as the emission angle is increased. Analysis of a clean sample shows that the layer-dependent composition of the un-oxidized HEA does not change significantly with depth; that is, the same concentration extends from the bulk to the surface. Thus, the increase in the ratio at higher angles means that the oxide species are confined to the surface selvage (ca. 0.5 nm), and there is no indication of oxygen diffusing into the subsurface region. Moreover, the lower exposure (10 L) data reveal that the initial oxidation of Cr and Fe is almost entirely within the first atomic layer, while Ni remains essentially unperturbed. At the higher exposure (1000 L), while surface Ni remains unreacted with oxygen, the oxidation of the other three metals (Cr, Fe, and Co) is limited to the surface only. Additionally, comparison of a clean sample before and after exposure to oxygen shows that the elemental composition of the surface region remains unchanged after oxidation. The angle-dependent XPS results support the use of a metal slab with adsorbed O atoms to model the HEA surface under lightly oxidizing conditions.

## Conclusions

In summary, we have theoretically analyzed the surface reactivity of a quaternary equimolar HEA, CoCrFeNi, using a surface model derived from an optimally constructed finite-sized SCRAPs supercell approximating the bulk HEA. The results suggest that the HEA surface should exist in a partially oxidized state when it is exposed to an acidic solution in the HER potential regime. On such a surface, the more reactive sites are occupied by oxygen, but a high percentage of sites would remain open, where hydrogen adsorption is modulated by the adsorbed O atoms to a level that suggests a higher HER activity than the individual metals. Electrochemical testing of this HEA of 3d base metals in a 0.5 M H_2_SO_4_ aqueous solution confirms it to be highly active for the HER. The overpotential, as measured at 1 mA/cm^2^, is only 60 mV larger than that of Pt. The HEA is more stable than the individual base metals and shows only minor dissolution after cycling between 0 and −0.45 V for 1000 times without significant loss of activity. The shifts in the binding energy of the primary 2p_3/2_ peaks of the component elements in XPS spectra show that the HEA preferentially oxidizes to extents that are in the order of Cr ≫ Fe > Co, with no evidence of Ni oxidation even after 1000 L of O_2_ exposure. The resistance of Ni to oxidation also agrees with our theoretical results.

The experimental confirmation suggests that our approach is effective for the analysis of the catalytic activity of random alloy surfaces. We further anticipate that slight strengthening of H adsorption should make this four-component random alloy still more active for HER. This could perhaps be best accomplished by a small increase of the concentration of Ni, which would lessen the extent to which the alloy surface is oxidized and shift its reactivity in the direction of Pt. The possibility of dialing in on desired catalytic activity with theoretically predicted random alloy compositions points to a systematic approach for guiding the substitution of precious metals with other, more earth-abundant metals, and to a need to better understand the role of electronic structure in complex alloys in catalysis.

## Supporting Information Available

Images of a four-layer (4√2 × 4√2) slab model of the (100) facet of the HEA and the reverse side of the four-layer (6 × 6) slab model of the (111) facet of the HEA; EBSD image of a typical HEA sample; Tafel plot; and atomic coordinates (in bulk positions) of the slab models of the (111) and (100) facets of the HEA (PDF)
