# Supplementary material for: CoCrFeNi High-Entropy Alloy as an Enhanced Hydrogen Evolution Catalyst in an Acidic Solution
Source: J Phys Chem C Nanomater Interfaces. 2021 Aug 3;125(31):17008–18. doi: 10.1021/acs.jpcc.1c03646 (PMC8392348; doi:10.1021/acs.jpcc.1c03646)
Supplement: Supplementary file 1 — jp1c03646_si_001.pdf [file jp1c03646_si_001.pdf]

CoCrFeNi High Entropy Alloy as Enhanced Hydrogen Evolution Catalyst in an Acidic Solution

Frank McKay,[1] Yuxin Fang,[2] Orhan Kizilkaya,[3] Prashant Singh,[4] Duane D. Johnson,[4,5] Amitava Roy,[3] David Young,[1] Phillip T. Sprunger,[1] John C. Flake,[2] William A. Shelton,[2] Ye Xu[2]*

[1] Department of Physics and Astronomy, Louisiana State University, Baton Rouge, LA 70803, USA

[2] Cain Department of Chemical Engineering, Louisiana State University, Baton Rouge, LA 70803, USA

[3] Center for Advanced Microstructures and Devices, Louisiana State University, Baton Rouge, LA 70803, USA

[4] Ames Laboratory, United States Department of Energy, Ames, IA 50011, USA

[5] Department of Materials Science and Engineering, Iowa State University, Ames, IA 50011, USA

Supporting Information

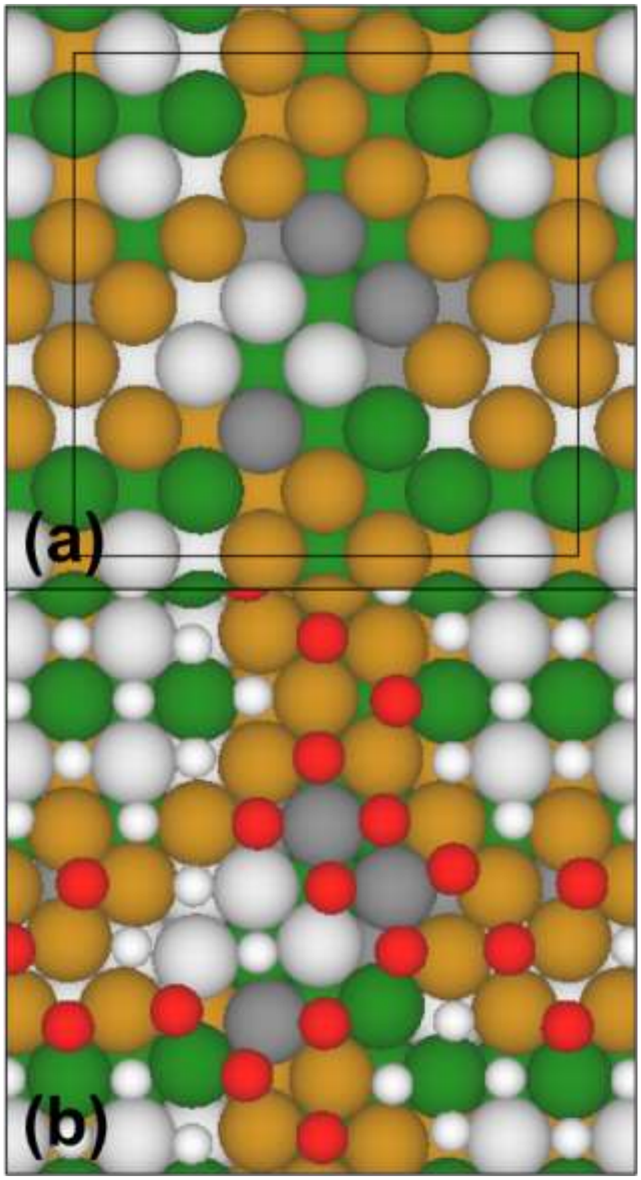

Figure S1. A (4√2×4√2) surface model for the (100) facet of the CoCrFeNi HEA. (a) Clean surface with surface unit cell outlined (black line); (b) extent to which the surface will be oxidized at 0 V vs. SHE and pH 0, with all remaining 4-fold open sites occupied by H atoms. Color code: Co = green; Cr = dark grey; Fe = yellow; Ni = white; O = red; H = white (small).

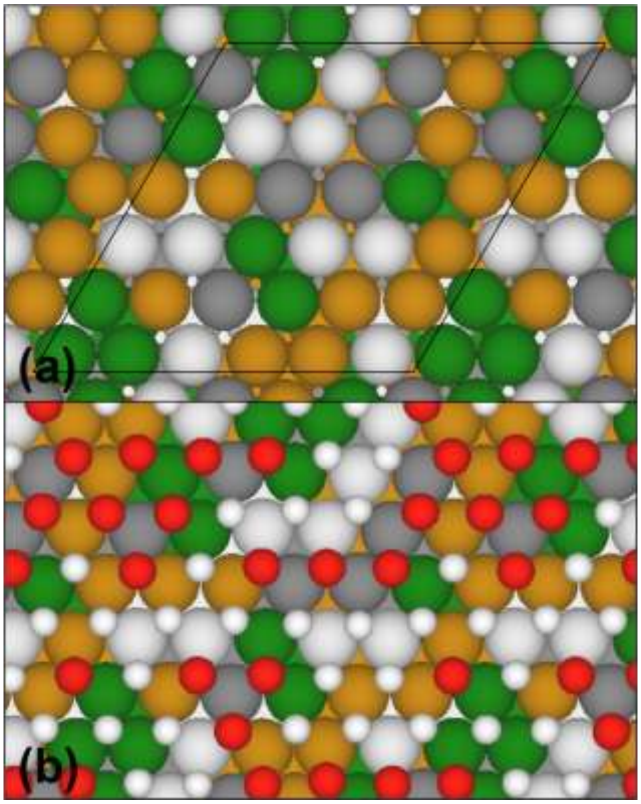

Figure S2. The reverse side of the (6×6) (111) surface model for the CoCrFeNi HEA shown in Figure 2. (a) Clean surface with surface unit cell outlined (black line); (b) extent to which the surface will be oxidized at 0 V vs. SHE and pH 0, with all remaining *fcc* sites occupied by H atoms. Color code: Co = green; Cr = dark grey; Fe = yellow; Ni = white; O = red; H = white (small).

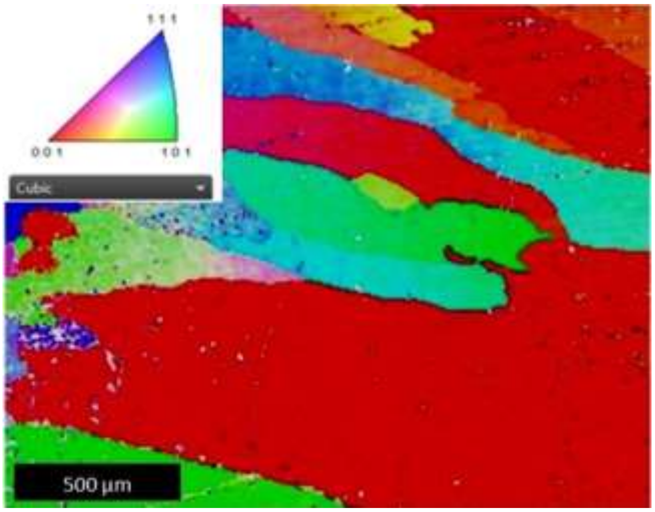

Figure S3. EBSD image of a typical as-cut CoCrFeNi HEA sample. Inset indicates the color scale of bulk crystal grain orientation.

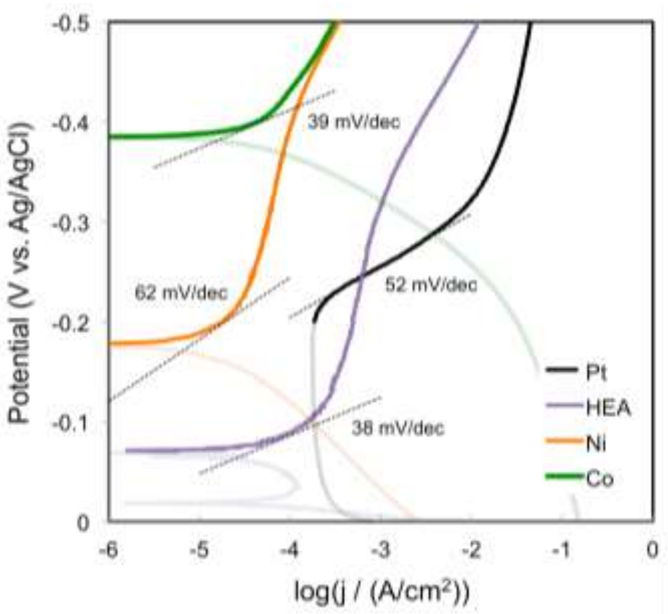

Figure S4. Tafel plot derived from the results in Figure 7. Dashed lines illustrate the Tafel slopes fitted for the Pt, HEA, Ni, and Co electrodes.

Atomic coordinates (in bulk positions) for the 4-layer (6×6) slab model of the (111) facet of the CoCrFeNi HEA

```
Co    0.555555556 0.277777777 0.333333333
Co    0.888888889 0.944444444 0.333333333
Co    0.388888889 0.111111111 0.333333333
Co    0.722222222 0.111111111 0.333333333
Co    0.722222222 0.944444444 0.333333333
Co    0.055555555 0.944444444 0.333333333
Co    0.555555556 0.777777777 0.333333333
Co    0.111111111 0.055555555 0.222222222
Co    0.111111111 0.722222222 0.222222222
Co    0.444444444 0.388888889 0.222222222
Co    0.777777777 0.722222222 0.222222222
Co    0.777777777 0.055555555 0.222222222
Co    0.277777777 0.555555556 0.222222222
Co    0.611111111 0.722222222 0.222222222
Co    0.111111111 0.555555556 0.222222222
Co    0.444444444 0.222222222 0.222222222
Co    0.777777777 0.222222222 0.222222222
Co    0.777777777 0.555555556 0.222222222
Co    0.333333333 0.500000000 0.111111111
Co    0.666666666 0.500000000 0.111111111
Co    0.166666666 0.000000000 0.111111111
Co    0.166666666 0.666666666 0.111111111
Co    0.833333333 0.333333333 0.111111111
Co    0.333333333 0.333333333 0.111111111
Co    0.333333333 0.666666666 0.111111111
Co    0.333333333 0.000000000 0.111111111
Co    0.666666666 0.333333333 0.111111111
Co    0.555555556 0.277777777 0.000000000
Co    0.888888889 0.277777777 0.000000000
Co    0.388888889 0.777777777 0.000000000
Co    0.388888889 0.444444444 0.000000000
Co    0.055555555 0.111111111 0.000000000
Co    0.555555556 0.111111111 0.000000000
Co    0.555555555 0.444444444 0.000000000
Co    0.555555555 0.777777778 0.000000000
Co    0.888888889 0.111111111 0.000000000
Cr    0.222222222 0.611111111 0.333333333
Cr    0.222222222 0.944444444 0.333333333
Cr    0.888888889 0.277777777 0.333333333
Cr    0.055555555 0.777777777 0.333333333
Cr    0.055555555 0.277777777 0.333333333
Cr    0.055555555 0.611111111 0.333333333
Cr    0.222222222 0.444444444 0.333333333
Cr    0.222222222 0.111111111 0.333333333
Cr    0.555555556 0.111111111 0.333333333
Cr    0.555555556 0.444444444 0.333333333
Cr    0.888888889 0.777777777 0.333333333
Cr    0.444444444 0.055555555 0.222222222
Cr    0.777777777 0.388888889 0.222222222
```

Cr	0.611111111	0.222222222	0.222222222
Cr	0.944444444	0.888888889	0.222222222
Cr	0.277777777	0.388888889	0.222222222
Cr	0.611111111	0.388888889	0.222222222
Cr	0.944444444	0.388888889	0.222222222
Cr	0.111111111	0.888888889	0.222222222
Cr	0.777777777	0.888888889	0.222222222
Cr	0.000000000	0.166666666	0.111111111
Cr	0.500000000	0.666666666	0.111111111
Cr	0.833333333	0.000000000	0.111111111
Cr	0.166666666	0.833333333	0.111111111
Cr	0.833333333	0.166666666	0.111111111
Cr	0.833333333	0.500000000	0.111111111
Cr	0.000000000	0.333333333	0.111111111
Cr	0.222222222	0.944444444	0.000000000
Cr	0.722222222	0.444444444	0.000000000
Cr	0.055555555	0.777777777	0.000000000
Cr	0.388888889	0.611111111	0.000000000
Cr	0.055555555	0.944444444	0.000000000
Cr	0.055555555	0.277777777	0.000000000
Cr	0.222222222	0.111111111	0.000000000
Fe	0.222222222	0.277777777	0.333333333
Fe	0.888888889	0.611111111	0.333333333
Fe	0.055555555	0.111111111	0.333333333
Fe	0.388888889	0.944444444	0.333333333
Fe	0.388888889	0.277777777	0.333333333
Fe	0.722222222	0.611111111	0.333333333
Fe	0.222222222	0.777777777	0.333333333
Fe	0.888888889	0.444444444	0.333333333
Fe	0.444444444	0.722222222	0.222222222
Fe	0.277777777	0.222222222	0.222222222
Fe	0.611111111	0.555555555	0.222222222
Fe	0.611111111	0.888888889	0.222222222
Fe	0.944444444	0.222222222	0.222222222
Fe	0.944444444	0.555555555	0.222222222
Fe	0.611111111	0.055555555	0.222222222
Fe	0.444444444	0.555555555	0.222222222
Fe	0.333333333	0.166666666	0.111111111
Fe	0.666666666	0.833333333	0.111111111
Fe	0.500000000	0.333333333	0.111111111
Fe	0.500000000	0.000000000	0.111111111
Fe	0.833333333	0.666666666	0.111111111
Fe	0.500000000	0.500000000	0.111111111
Fe	0.500000000	0.166666666	0.111111111
Fe	0.000000000	0.000000000	0.111111111
Fe	0.000000000	0.666666666	0.111111111
Fe	0.666666666	0.666666666	0.111111111
Fe	0.666666666	0.000000000	0.111111111
Fe	0.555555555	0.944444444	0.000000000
Fe	0.888888889	0.611111111	0.000000000
Fe	0.722222222	0.111111111	0.000000000
Fe	0.722222222	0.777777777	0.000000000
Fe	0.055555555	0.444444444	0.000000000
Fe	0.722222222	0.277777777	0.000000000
Fe	0.722222222	0.944444444	0.000000000
Fe	0.222222222	0.777777777	0.000000000
Fe	0.222222222	0.444444444	0.000000000

Fe	0.888888889	0.444444444	0.000000000
Fe	0.888888889	0.777777777	0.000000000
Ni	0.555555556	0.611111111	0.333333333
Ni	0.555555556	0.944444444	0.333333333
Ni	0.388888889	0.444444444	0.333333333
Ni	0.388888889	0.777777777	0.333333333
Ni	0.722222222	0.777777777	0.333333333
Ni	0.722222222	0.444444444	0.333333333
Ni	0.055555555	0.444444444	0.333333333
Ni	0.388888889	0.611111111	0.333333333
Ni	0.722222222	0.277777777	0.333333333
Ni	0.888888889	0.111111111	0.333333333
Ni	0.111111111	0.388888889	0.222222222
Ni	0.277777777	0.888888889	0.222222222
Ni	0.277777777	0.722222222	0.222222222
Ni	0.277777777	0.055555555	0.222222222
Ni	0.944444444	0.055555555	0.222222222
Ni	0.944444444	0.722222222	0.222222222
Ni	0.111111111	0.222222222	0.222222222
Ni	0.444444444	0.888888889	0.222222222
Ni	0.000000000	0.833333333	0.111111111
Ni	0.000000000	0.500000000	0.111111111
Ni	0.333333333	0.833333333	0.111111111
Ni	0.666666666	0.166666666	0.111111111
Ni	0.166666666	0.333333333	0.111111111
Ni	0.166666666	0.166666666	0.111111111
Ni	0.166666666	0.500000000	0.111111111
Ni	0.500000000	0.833333333	0.111111111
Ni	0.833333333	0.833333333	0.111111111
Ni	0.222222222	0.611111111	0.000000000
Ni	0.222222222	0.277777777	0.000000000
Ni	0.555555555	0.611111111	0.000000000
Ni	0.888888889	0.944444444	0.000000000
Ni	0.388888889	0.111111111	0.000000000
Ni	0.388888889	0.944444444	0.000000000
Ni	0.388888889	0.277777777	0.000000000
Ni	0.722222222	0.611111111	0.000000000
Ni	0.055555555	0.611111111	0.000000000

Atomic coordinates (in bulk positions) for the 4-layer (4√2×4√2) slab model of the (100) facet of the CoCrFeNi HEA

```
Co    0.00000000  0.00000000  0.00000000
Co    0.00000000  0.75000000  0.00000000
Co    0.25000000  0.50000000  0.00000000
Co    0.37500000  0.37500000  0.00000000
Co    0.75000000  0.00000000  0.00000000
Co    0.75000000  0.75000000  0.00000000
Co    0.12500000  0.00000000  0.07856742
Co    0.12500000  0.75000000  0.07856742
Co    0.37500000  0.25000000  0.07856742
Co    0.37500000  0.50000000  0.07856742
Co    0.62500000  0.50000000  0.07856742
Co    0.87500000  0.00000000  0.07856742
Co    0.87500000  0.75000000  0.07856742
Co    0.12500000  0.12500000  0.15713484
Co    0.12500000  0.62500000  0.15713484
Co    0.12500000  0.87500000  0.15713484
Co    0.37500000  0.37500000  0.15713484
Co    0.50000000  0.00000000  0.15713484
Co    0.50000000  0.25000000  0.15713484
Co    0.50000000  0.50000000  0.15713484
Co    0.50000000  0.75000000  0.15713484
Co    0.62500000  0.12500000  0.15713484
Co    0.62500000  0.62500000  0.15713484
Co    0.62500000  0.87500000  0.15713484
Co    0.87500000  0.12500000  0.15713484
Co    0.87500000  0.62500000  0.15713484
Co    0.87500000  0.87500000  0.15713484
Co    0.00000000  0.12500000  0.23570226
Co    0.00000000  0.87500000  0.23570226
Co    0.25000000  0.12500000  0.23570226
Co    0.25000000  0.87500000  0.23570226
Co    0.62500000  0.25000000  0.23570226
Co    0.75000000  0.12500000  0.23570226
Co    0.75000000  0.87500000  0.23570226
Cr    0.00000000  0.25000000  0.00000000
Cr    0.00000000  0.50000000  0.00000000
Cr    0.12500000  0.62500000  0.00000000
Cr    0.25000000  0.25000000  0.00000000
Cr    0.37500000  0.12500000  0.00000000
Cr    0.37500000  0.87500000  0.00000000
Cr    0.62500000  0.37500000  0.00000000
Cr    0.62500000  0.62500000  0.00000000
Cr    0.75000000  0.25000000  0.00000000
Cr    0.75000000  0.50000000  0.00000000
Cr    0.87500000  0.62500000  0.00000000
Cr    0.25000000  0.62500000  0.07856742
Cr    0.50000000  0.12500000  0.07856742
Cr    0.50000000  0.87500000  0.07856742
Cr    0.00000000  0.50000000  0.15713484
Cr    0.37500000  0.62500000  0.15713484
Cr    0.62500000  0.37500000  0.15713484
```

Cr	0.75000000	0.50000000	0.15713484
Cr	0.37500000	0.25000000	0.23570226
Cr	0.50000000	0.62500000	0.23570226
Cr	0.62500000	0.50000000	0.23570226
Fe	0.12500000	0.12500000	0.00000000
Fe	0.12500000	0.37500000	0.00000000
Fe	0.12500000	0.87500000	0.00000000
Fe	0.37500000	0.62500000	0.00000000
Fe	0.50000000	0.00000000	0.00000000
Fe	0.50000000	0.75000000	0.00000000
Fe	0.87500000	0.12500000	0.00000000
Fe	0.87500000	0.37500000	0.00000000
Fe	0.87500000	0.87500000	0.00000000
Fe	0.00000000	0.37500000	0.07856742
Fe	0.50000000	0.62500000	0.07856742
Fe	0.62500000	0.00000000	0.07856742
Fe	0.62500000	0.75000000	0.07856742
Fe	0.75000000	0.37500000	0.07856742
Fe	0.00000000	0.00000000	0.15713484
Fe	0.00000000	0.75000000	0.15713484
Fe	0.25000000	0.25000000	0.15713484
Fe	0.37500000	0.12500000	0.15713484
Fe	0.37500000	0.87500000	0.15713484
Fe	0.75000000	0.00000000	0.15713484
Fe	0.75000000	0.75000000	0.15713484
Fe	0.00000000	0.37500000	0.23570226
Fe	0.00000000	0.62500000	0.23570226
Fe	0.12500000	0.25000000	0.23570226
Fe	0.12500000	0.50000000	0.23570226
Fe	0.25000000	0.62500000	0.23570226
Fe	0.37500000	0.00000000	0.23570226
Fe	0.37500000	0.75000000	0.23570226
Fe	0.50000000	0.12500000	0.23570226
Fe	0.50000000	0.87500000	0.23570226
Fe	0.62500000	0.00000000	0.23570226
Fe	0.62500000	0.75000000	0.23570226
Fe	0.75000000	0.37500000	0.23570226
Fe	0.75000000	0.62500000	0.23570226
Fe	0.87500000	0.25000000	0.23570226
Fe	0.87500000	0.50000000	0.23570226
Ni	0.25000000	0.00000000	0.00000000
Ni	0.25000000	0.75000000	0.00000000
Ni	0.50000000	0.25000000	0.00000000
Ni	0.50000000	0.50000000	0.00000000
Ni	0.62500000	0.12500000	0.00000000
Ni	0.62500000	0.87500000	0.00000000
Ni	0.00000000	0.12500000	0.07856742
Ni	0.00000000	0.62500000	0.07856742
Ni	0.00000000	0.87500000	0.07856742
Ni	0.12500000	0.25000000	0.07856742
Ni	0.12500000	0.50000000	0.07856742
Ni	0.25000000	0.12500000	0.07856742
Ni	0.25000000	0.37500000	0.07856742
Ni	0.25000000	0.87500000	0.07856742
Ni	0.37500000	0.00000000	0.07856742
Ni	0.37500000	0.75000000	0.07856742
Ni	0.50000000	0.37500000	0.07856742

Ni	0.62500000	0.25000000	0.07856742
Ni	0.75000000	0.12500000	0.07856742
Ni	0.75000000	0.62500000	0.07856742
Ni	0.75000000	0.87500000	0.07856742
Ni	0.87500000	0.25000000	0.07856742
Ni	0.87500000	0.50000000	0.07856742
Ni	0.00000000	0.25000000	0.15713484
Ni	0.12500000	0.37500000	0.15713484
Ni	0.25000000	0.00000000	0.15713484
Ni	0.25000000	0.50000000	0.15713484
Ni	0.25000000	0.75000000	0.15713484
Ni	0.75000000	0.25000000	0.15713484
Ni	0.87500000	0.37500000	0.15713484
Ni	0.12500000	0.00000000	0.23570226
Ni	0.12500000	0.75000000	0.23570226
Ni	0.25000000	0.37500000	0.23570226
Ni	0.37500000	0.50000000	0.23570226
Ni	0.50000000	0.37500000	0.23570226
Ni	0.87500000	0.00000000	0.23570226
Ni	0.87500000	0.75000000	0.23570226
